# Lentiviral Vector Production Titer Is Not Limited in HEK293T by Induced Intracellular Innate Immunity

**DOI:** 10.1016/j.omtm.2019.11.021

**Published:** 2019-12-24

**Authors:** Carolina B. Ferreira, Rebecca P. Sumner, Maria T. Rodriguez-Plata, Jane Rasaiyaah, Richard S. Milne, Adrian J. Thrasher, Waseem Qasim, Greg J. Towers

**Affiliations:** 1 Molecular and Cellular Immunology Unit, Great Ormond Street Institute of Child Health, University College London, London WC1N 1EH, UK; 2 Division of Infection and Immunity, University College London, London WC1E 6BT, UK; 3 Great Ormond Street Hospital for Children NHS Foundation Trust, London WC1N 1EH, UK

**Keywords:** Lentiviral vectors, innate immunity, HEK293T, vector titer, SV40 large T-antigen

## Abstract

Most gene therapy lentiviral vector (LV) production platforms employ HEK293T cells expressing the oncogenic SV40 large T-antigen (TAg) that is thought to promote plasmid-mediated gene expression. Studies on other viral oncogenes suggest that TAg may also inhibit the intracellular autonomous innate immune system that triggers defensive antiviral responses upon detection of viral components by cytosolic sensors. Here we show that an innate response can be generated after HIV-1-derived LV transfection in HEK293T cells, particularly by the transgene, yet, remarkably, this had no effect on LV titer. Further, overexpression of DNA sensing pathway components led to expression of inflammatory cytokine and interferon (IFN) stimulated genes but did not result in detectable IFN or CXCL10 and had no impact on LV titer. Exogenous IFN-β also did not affect LV production or transduction efficiency in primary T cells. Additionally, manipulation of TAg did not affect innate antiviral responses, but stable expression of TAg boosted vector production in HEK293 cells. Our findings demonstrate a measure of innate immune competence in HEK293T cells but, crucially, show that activation of inflammatory signaling is uncoupled from cytokine secretion in these cells. This provides new mechanistic insight into the unique suitability of HEK293T cells for LV manufacture.

## Introduction

Lentiviral vectors (LVs) are used in some of the most successful gene therapies including correction of inherited blood, immune, and metabolic disorders, as well as in immunotherapy using chimeric antigen receptor redirected T cells (CAR-T cells). Optimization of large-scale manufacturing platforms is critical for LV-mediated therapies to be implemented as standard of care. To date, clinical LVs are typically produced by transient plasmid-mediated co-expression of viral components in the HEK293T cell line.^1^, ^2^, ^3^, ^4^, ^5^ Lentivirus production is therefore a key limiting step in developing gene therapy as an efficient and cost-effective treatment. Innate immune activation caused by sensing of viral components could be an important factor in limiting maximal efficiency of LV production. Understanding whether vector components activate cell autonomous innate immune responses and whether these responses limit LV production is key to developing the most effective production strategies.

The cell-autonomous innate immune system includes germline-encoded sensors that patrol cellular compartments for pathogen-associated molecular patterns (PAMPs) or danger-associated molecular patterns (DAMPs). Upon triggering, sensors activate transcription factors including nuclear factor κB (NF-κB), interferon (IFN) regulatory factor 3 (IRF3), and activator protein 1 (AP1). The ensuing defensive responses include production of inflammatory cytokines, particularly type 1 IFN, and expression of IFN-stimulated antiviral genes that act directly to inhibit all stages of viral replication.^6^ Such responses may reduce LV yield through direct effects on producer cells. In addition, contamination of LV preparations with bioactive inflammatory cytokines may compromise their use in patient cells and impair downstream processes, for example, stem cell engraftment. LVs are derived from the human immunodeficiency virus (HIV), which induces strong innate and adaptive responses on infection.^7^^,^^8^ In the current third generation vector configuration, viral accessory proteins have been deleted to reduce immunogenicity and minimize HIV sequence in the LV.^9^^,^^10^ However, these proteins counteract host antiviral responses, and thus multiply deleted LVs may be more stimulatory than wild-type HIV and may be more sensitive to defensive pathways.^11^, ^12^, ^13^, ^14^, ^15^

Moreover, introduction of foreign DNA into the cytoplasm of mammalian cells activates innate sensors and type 1 IFN secretion.^16^ The DNA sensor cyclic-GMP-AMP (cGAMP) synthase (cGAS) is a central intracellular sensor of DNA.^17^ Upon detection of DNA, activated cGAS produces the cyclic dinucleotide cGAMP, which in turn activates the endoplasmatic reticulum (ER)-associated membrane protein STING. Activated STING recruits TBK1 to activate transcription factors IRF3 and NF-κB through complex phosphorylation of both the transcription factors and STING itself.^18^ A key gene response on activation of cGAS is production of type 1 IFN.^16^^,^^19^ Importantly both cGAMP and IFN are secreted and can induce responses in neighboring cells.^20^ Furthermore, cGAMP can be incorporated into LV particles, stimulating defensive responses in target cells.^21^

The HEK293T cells used for LV production have been modified to stably express viral oncogenes, which have been associated with the dampening of intracellular innate responses. Parental HEK293 cells were transformed by introducing human adenovirus 5 (hAd5) DNA and then further modified by stable expression of the large T-antigen (TAg) of simian virus 40 (SV40) to generate HEK293T cells.^22^^,^^23^ The TAg and adenovirus E1A expressed in HEK293T inactivate the cellular tumor suppressors p53 and retinoblastoma^24^ and also inhibit antiviral responses by interfering with IRF3 or IFN-dependent transcription downstream of RNA and DNA sensor activation.^25^, ^26^, ^27^, ^28^

In this study, we investigated the intracellular innate immune response in HEK293T during LV production and its impact on LV yield. We show that an innate immune response can be activated in HEK293T. However, cytokines including type 1 IFN are not detectable, vector production is not significantly impacted, and transduction efficiency of resulting LV preparations is not reduced on IFN-sensitive monocytic THP-1 cells, or primary human T cells. We further show that TAg expression improves LV production but does not affect activation of innate responses by DNA sensing. Our findings improve understanding of the impact of innate immune activation on LV production and could guide optimization of current vector configurations, inform next generation vector design, and help circumvent a major bottleneck in widespread deployment of LV for gene therapy.

## Results

### Activation of Innate Immune Signaling in HEK293T Does Not Impact LV Yield

**Figure 1:**
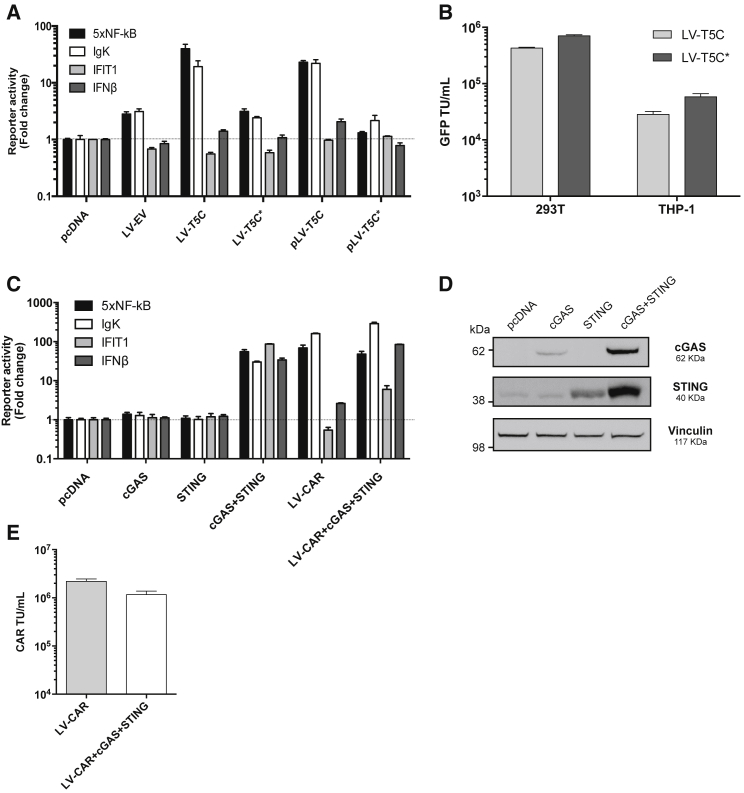
Production of Gene Therapy LVs in HEK293T Triggers NF-κB Activation but Does Not Impact LV Yield (A) HEK293T cells were transfected with the indicated firefly luciferase reporter constructs, pRL-TK *Renilla* luciferase, and empty pcDNA3 as a control or LV constructs including VSV-G envelope encoding pMD2.G, pCMV-dR8.74 Gag-Pol expression plasmid, and a genome plasmid bearing IRES-GFP as follows: LV-EV is LV genome bearing empty vector, LV-T5C encodes full-length humanized TRIM5-CypA chimera, LV-T5C* encodes full-length humanized TRIM5-CypA chimera with mutated start methionines at positions 1 and 47. pLV-T5C and pLV-T5C* denote genome plasmids alone without pMD2.G or pCMV-dR8.74 co-transfection. Firefly luciferase values were normalized to *Renilla* luciferase values. Data represent mean fold change in reporter activity ± SD (n = 3) 48 h post transfection presented relative to cells transfected with an equivalent amount of pcDNA3. (B) Culture supernatants from (A) were harvested at 48 h and mean viral titers ± SD of biological replicates (n = 2) were determined in duplicate in HEK293T and THP-1 cells by enumerating GFP-positive cells. TU, transducing units. (C) HEK293T cells were transfected with the indicated firefly luciferase reporter constructs, pRL-TK *Renilla* luciferase, and pcDNA-based expression plasmids encoding cGAS and/or STING as shown and/or a LV encoding a chimeric antigen receptor (CAR), as well as pCMV-dR8.74 and pMD2.G (LV-CAR). Mean fold change in reporter activity ± SD (n = 3) was assessed 48 h later using a dual-luciferase reporter assay and is presented relative to cells transfected with an equivalent amount of pcDNA. (D) Cell extracts from transfected HEK293T cells were subjected to immunoblot detecting cGAS or STING or vinculin as loading control. Molecular mass markers are shown. (E) Culture supernatants from (C) containing infectious LV CAR were harvested at 48 h and viral titers ± SD of biological replicates (n = 2) were determined in duplicate in HEK293T cells by qPCR with CAR encoding LV-specific probes.

We first tested whether production of LV by transient transfection induces an innate immune response in HEK293T producer cells. HEK293T cells were co-transfected with LV producing plasmids and a panel of luciferase-encoding reporter constructs sensitive to activation by a variety of innate immune responses including type 1 IFN and transcription factors NF-κB and IRF3. We found that production of LV encoding GFP weakly induced synthetic NF-κB-sensitive reporter constructs bearing either NF-κB p50/p65 binding sites (5×NF-κB) or NF-κB binding sites from the immunoglobulin kappa light chain (Igκ) promoter (LV-EV, Figure 1A). By contrast, production of LVs encoding the TRIM5α-cyclophilin A antiviral fusion (T5C) protein strongly induced both of these NF-κB-sensitive reporters (LV-T5C, Figure 1A) consistent with the documented activation of NF-κB inflammatory signaling by TRIM5α.^29^ Neither LV activated the IFN-β reporter, or the IRF3/IFN-responsive IFIT1-reporter (Figure 1A).

Mutation of the TRIM5-CypA start codon reduced activation of the NF-κB-sensitive reporters (T5C*, Figure 1A) to the level activated by empty vector, while transfection of the TRIM5-CypA-encoding LV genome alone (pLV-T5C) activated the NF-κB-sensitive reporters to equivalent levels as LV-T5C. Thus, the LV packaging and Env constructs made only minimal contribution to the NF-κB activation seen with the TRIM5-CypA vector. Further, the observed fold change in NF-κB-sensitive reporter activation was representative of a true activation of these reporters as expression of the constitutively expressed TK *Renilla* reporter, to which the data was normalized, was unchanged by LV-T5C transfection (Figure S1A).

We next asked whether this potent NF-κB activation affected vector yield. We found that the TRIM5-CypA vector and the LV-T5C* vector, which does not express TRIM5-CypA protein, had similar infectious titers, with the TRIM5-CypA-encoding LV giving slightly lower infectivities on both HEK293T cells and THP-1 monocyte-like cells (Figure 1B). Thus similar infectivity is seen even in cells that are less permissive than HEK293T to HIV-1-derived LV such as the THP-1 cells. In addition, THP-1 respond to inflammatory cytokines, such as type 1 IFN, further reducing their permissivity to infection.^30^, ^31^, ^32^

These data suggest that innate immune activation of HEK293T in response to LV production does not strongly impact LV yield. However, it is likely that novel gene therapies will deploy constructs that directly activate innate immune and inflammatory responses. For example, gene delivery of type 1 IFN itself has been proposed in anti-tumor strategies^33^^,^^34^ and modified T cell receptors such as CARs are likely to activate signaling pathways. We therefore set out to measure the effect of an artificial and potent innate immune response on LV production in HEK293T cells.

First, we activated a DNA sensing response by co-transfection of plasmids encoding the DNA sensor cGAS and the ER-associated signaling protein STING. As expected, overexpression of cGAS and STING led to the activation of NF-κB-sensitive and IFIT1 and IFN-β promoters (Figure 1C). There was no response when either cGAS or STING was expressed alone. Immunoblotting of unmodified HEK293T cells revealed low levels of endogenous STING, likely unable to facilitate activation of the luciferase reporters when cGAS alone was overexpressed (Figures 1C and 1D)^35^ and endogenous cGAS was below immunoblot detection levels (Figure 1D).

We also tested a therapeutic LV encoding a CAR (LV-CAR).^36^ Comparison of LV-CAR (Figure 1C, LV-CAR) with an empty vector (Figure 1A, LV-EV), suggests that LV-CAR activated NF-κB-responsive reporters, consistent with a signaling response being induced by overexpression of a modified T cell receptor molecule. IFN-β and IFIT1 promoters were not activated by LV-CAR expression. Co-transfection of LV-CAR components, and cGAS/STING together, induced activation of all four reporters (Figure 1C), but importantly, this maximal innate immune activation had only minimal impact on LV titer when HEK293T cells were used as target cells (Figure 1E). Again, expression of the constitutive TK *Renilla* reporter was not significantly impacted by expression of cGAS/STING or LV-CAR (Figure S1B).

In order to consider the general relevance of our findings, we tested two independent HEK293T cell lines. Titers of a GFP-encoding LV were found to be comparable between our HEK293T producer cells and HEK293FT (obtained from Thermo Fisher Scientific) and HEK293T JL cells (obtained from the laboratory of Jeremy Luban, University of Massachusetts Medical School, USA) (compare Figure 4E and Figure S1C). As with our HEK293T producer cells, neither HEK293FT or HEK293T JL cells showed significant activation of an NF-κB-sensitive promoter after transfection of cGAS and STING alone under the conditions tested, but both showed considerable activation following co-transfection of these components (Figure S1D). The HEK293T JL gave the largest activation, possibly due to their superior transfection efficiency and/or expression evidenced by greater *Renilla* luciferase values expressed from the control plasmid (Figure S1E).

Together, these data suggest that LV production in HEK293T cells can induce an inflammatory transcriptional response, with the magnitude of the response depending on the transgene. Crucially, the vector components themselves do not trigger strong responses. However, although some transgenes activate signaling, this has minimal, if any, impact on the vector production, or on the titer of the vector produced, at least for the target cells used here.

### Activation of DNA Sensing in HEK293T Elicits a Transcriptional Pro-inflammatory Response but Does Not Result in the Secretion of Measureable Type 1 IFN or CXCL10

**Figure 2:**
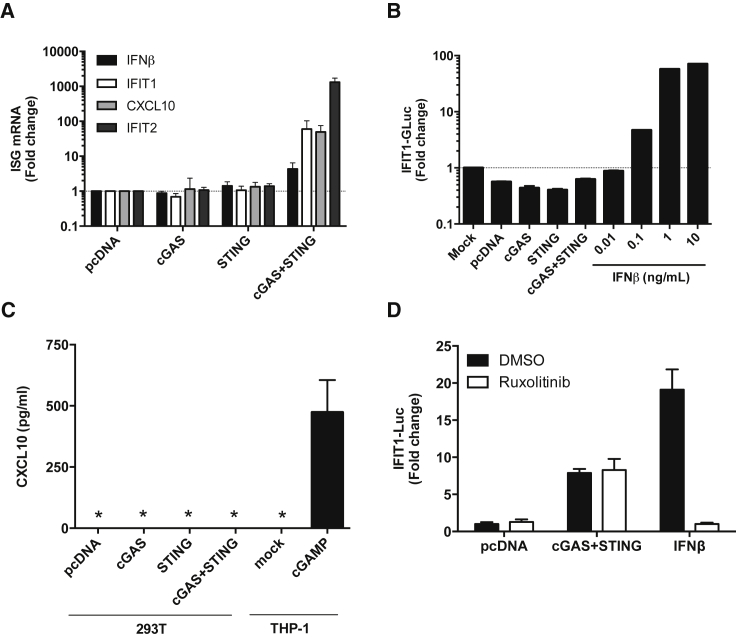
Activation of DNA Sensing in HEK293T Elicits a Transcriptional Pro-inflammatory Response but Does Not Result in the Secretion of Measureable Type 1 IFN or CXCL10 (A) qRT-PCR for the indicated genes was performed on RNA extracted from HEK293T 48 h after transfection with the indicated pcDNA-based expression plasmids. Bars represent means ± SD (n = 2) of biological replicates relative to cells transfected with an equivalent amount of pcDNA. (B) Supernatants from HEK293T cells transfected with the indicated plasmids were harvested after 48 h, filtered, and incubated with THP-1 reporter cells bearing Gaussia Luciferase under the control of the endogenous IFIT1 promoter. Luciferase was measured after 24 h as a measure of IFN-β. Exogenous IFN-β (0.01–10 ng/mL) was used as a control. (C) CXCL10 in the supernatant of HEK293T 48 h post transfection with the indicated plasmids was measured by ELISA. Supernatant from THP-1 cells stimulated for 24 h with cGAMP (1 μg/mL) was used as a positive control. * indicates below the limit of detection. (D) HEK293T cells were co-transfected with the indicated pcDNA-based expression plasmids, an IFIT1 firefly luciferase reporter and pRL-TK Renilla luciferase. 24 h later, cells were incubated with DMSO vehicle or 2 μM ruxolitinib. At 48 h post transfection, luciferase activity was measured and firefly luciferase values were normalized to *Renilla* luciferase values. Bars represent mean fold induction of IFIT1 luc activity (±SD, n = 3) presented relative to cells transfected with an equivalent amount of pcDNA. Cells were treated with 10 ng/mL exogenous IFN-β as a control.

Having demonstrated that HEK293T can respond to activation of DNA sensing pathways, we sought to understand the nature of this response and the degree of IFN production. We found that cGAS/STING expression in HEK293T strongly induced mRNA expression of various endogenous IFN-stimulated genes (ISGs) including *IFIT1*, *CXCL10*, and *IFIT2.* Importantly, these ISGs were not induced by either cGAS or STING expression alone, consistent with a requirement for activation of transfected STING by cGAMP produced by transfected cGAS under these conditions (Figure 2A). This experiment demonstrates that the IRF3- and NF-κB- dependent signaling pathways downstream of STING are intact in HEK293T and that these cells can induce ISG expression. ISG induction might be due to direct activation through recruitment of NF-κB and IRF3 to the promoters, the so-called first line response, or it may be due to induction of type 1 IFN expression and induction of ISG expression via activation of the type 1 IFN receptor and subsequent signaling.

To test whether the HEK293T cells produced detectable IFN upon activation by cGAS/STING expression, we added filtered supernatants from cGAS/STING transfected HEK293T to THP-1 encoding an IFN-sensitive IFIT1-luciferase reporter.^37^ We observed no luciferase induction (Figure 2B), consistent with the low level activation of *IFN-β* mRNA expression on cGAS/STING expression (Figure 2A). The THP-1 reporter cells are sensitive to very low levels of IFN and as little as 0.1 ng/mL recombinant IFN-β activated reporter activity (Figure 2B). Furthermore, mRNA levels of *CXCL10* (a proinflammatory chemokine and ISGs) were upregulated by up to ∼50-fold (Figure 2A). However, we could not detect CXCL10 protein in supernatants by ELISA at 48 h (Figure 2C). As a positive control, CXCL10 was readily detectable in supernatant from cGAMP-treated THP-1 cells 24 h after stimulation (Figure 2C). To inhibit the effects of type 1 IFN production, we used the Janus kinase (JAK) inhibitor ruxolitinib. Type 1 IFN receptor signaling is JAK-dependent and ruxolitinib-sensitive and thus ruxolitinib distinguishes between IFN dependent from direct activation of ISG expression.^38^ Critically, activation of the IFIT1-luc reporter by cGAS/STING was not reduced in the presence of ruxolitinib. This reveals that activation of IFIT1-luc is not dependent on IFN production in HEK293T cells (Figure 2D) consistent with failure to measure active IFN in the HEK293T supernatants. Failure to reduce IFIT1-luc expression with ruxolitinib suggests that cGAS/STING expression does not cause type 1 IFN production but rather activates the IFIT1 promoter through activation of IRF3. Importantly, activation of IFIT1-luc by IFN-β was effectively inhibited by ruxolitinib treatment as a positive control (Figure 2D).

Our results demonstrate that NF-κB, IFN-β, and IRF3 signaling pathways can be activated in HEK293T cells, resulting in an increase in proinflammatory cytokine mRNA expression. However, this innate immune activation does not result in detectable IFN-β or CXCL10 in the HEK293T supernatants 48 h after transfection, i.e., at the time that LV is typically harvested. These observations are consistent with the empirical identification of HEK293T cells as an effective production system for highly effective therapeutic LV because the activation of inflammatory signaling pathways is disconnected from the secretion of inflammatory cytokines in these cells. We assume that inflammatory cytokine secretion during vector manufacture would be detrimental because cytokine activity may interfere with several stages of virus production, as well as with transduction of target cells.

### Exogenous IFN-β Reduces LV Transduction Efficiency on Monocytic Cells but Not on HEK293T or Primary T Cells

**Figure 3:**
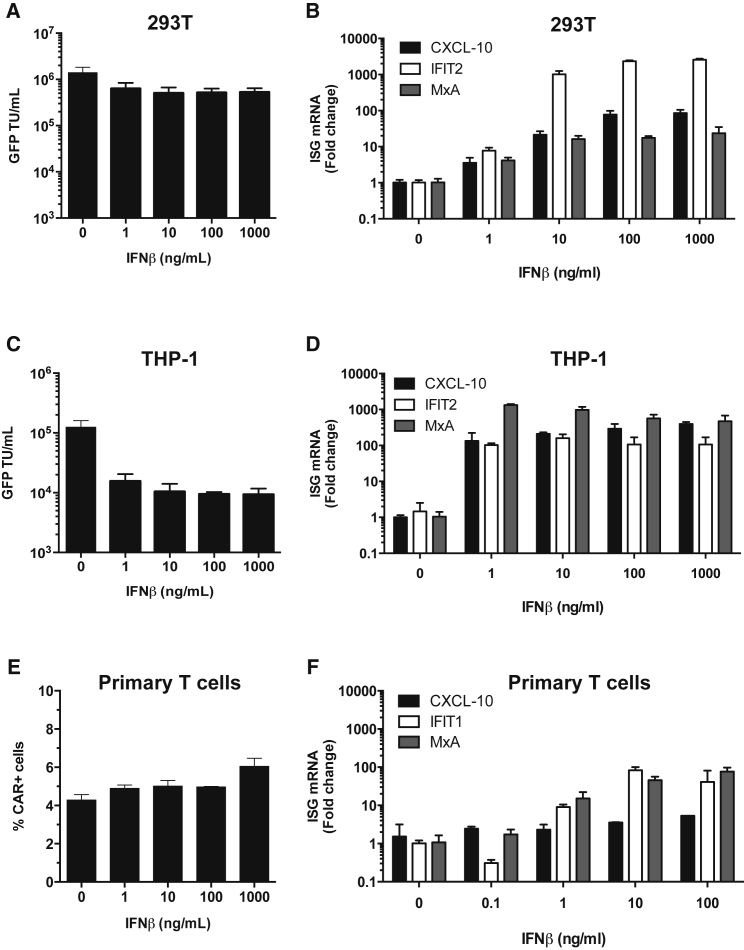
Exogenous IFN-β Reduces LV Transduction Efficiency on Monocytic Cells but Not on HEK293T or Primary T Cells (A and C) LV encoding GFP was used to infect (A) HEK293T or (C) THP-1 cells in the presence or absence of IFN-β titrations with infection measured by enumerating GFP-positive cells by flow cytometry (mean ± SD, n = 2 biological replicates). TU, transducing units. (B, D, and F) qRT-PCR for the indciated ISGs was performed after 24 h incubation of (B) HEK293T cells, (D) THP-1 cells, or (F) primary T cells stimulated with anti-CD3 and anti-CD28 beads with the indicated IFN-β concentrations (mean ± SD, n = 3 biological replicates). Measurements for each ISG were first normalized to GAPDH and then to mock-treated cells to generate a fold change. (E) Activated T cells were transduced with therapeutic CAR-encoding LV in the presence or absence of increasing amounts of IFN-β and transduction efficiency was assessed at day 5 by flow cytometry with an AffiniPure F(ab’) fragment antibody.

Previous reports have shown that pre-treatment of cultured human cells, or cell lines, with type 1 IFN mediates different levels of HIV-1 or LV inhibition ranging from severe inhibition in monocyte-derived macrophages, or in the monocytic line THP-1, to intermediate effects in primary CD4^+^ cells to minimal effects in immortalized T cell lines such as CEM or Jurkat.^31^^,^^32^^,^^39^ We found that LV infectivity was only slightly reduced by exogenous IFN-β when HEK293T cells were used as targets (Figure 3A). However, transduction of monocytic THP-1 cells was reduced by as much as 10-fold in the presence of as little as 1–10 ng/mL exogenous IFN-β (Figure 3C). Both HEK293T and THP-1 responded to IFN-β and expressed ISGs on IFN treatment, measured by quantitative reverse transcriptase PCR (qRT-PCR) (Figures 3B and 3D). A similar effect was observed when IFN-β was added at the time of vector plasmid transfection and IFN-β was added to the HEK293T producer line during vector production (Figure S2). These observations are consistent with the reported type 1 IFN induction of the antiviral protein IFITM3 in some cells, e.g., in THP-1 but not in HEK293T, which potently inhibits infection by vesicular stomatitis virus G (VSV-G) protein pseudotyped LV.^32^ We also measured the effect of IFN-β treatment of activated primary T cells on transduction levels by LV-CAR, measured by qPCR of LV genome (Figure 3E). We did this because primary T cells are the targets for therapeutic CAR expression. Importantly, the T cells responded to IFN-β by inducing ISG mRNA (Figure 3F) but infectivity of LV-CAR was not affected. We assume that ISGs with anti-LV activity were not induced in these cells in these experiments. In summary, IFN-β does not inhibit LV gene transfer or LV production in HEK293T cells, nor does it reduce LV transduction of primary T cells. However, the presence of IFN can influence transduction of other cell types, here exemplified by THP-1, although importantly IFN is not detectably produced by HEK293T, even when NF-κB and IRF3 are strongly activated (Figure 2).

### LV Production, but Not Innate Sensing, Is Influenced by SV40 Large TAg Expression

Finally, we sought to understand the role of the SV40 TAg in promoting high efficiency LV production in HEK293T despite innate immune activation mediated by, for example, TRIM5-CypA or cGAS/STING expression. A recent study proposed that the human adenovirus 5 (hAd5) *E1A* oncogene expressed in HEK293T disables STING through binding to an LXCXE motif and rendering these cells unresponsive to transfected DNA.^40^^,^^41^ Previous reports describing STING expression in certain transformed cell lines (including HEK293 but not in HEK293T^35^), along with the presence of an LXCXE binding motif in TAg, suggested that TAg may also be involved in the suppression of STING-dependent innate sensing pathways. We therefore considered whether TAg expression in HEK293T plays a role in reducing innate immune responses and enhancing LV production.

**Figure 4:**
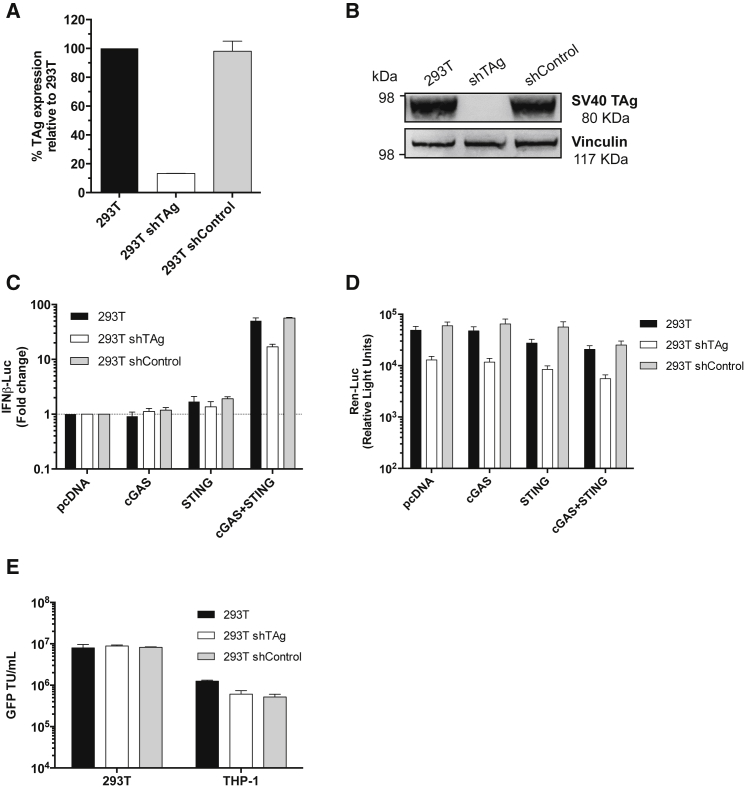
Large TAg Depletion in HEK293T Cells Does Not Impact cGAS/STING-Driven Activity of the IFN-β Luciferase Reporter or LV Production (A) SV40 large T-antigen (TAg) mRNA levels in HEK293T cells expressing TAg-specific shRNA or an shRNA control after 5 days of puromycin selection. TAg expression was first normalized to GAPDH and then to expression levels in the unmodified HEK293T cells to generate percentage relative expression. (B) TAg protein expression measured by immunoblot detecting TAg or vinculin as a loading control. Molecular mass markers are indicated. (C) Mean fold change in co-transfected IFN-β-luc reporter activity in HEK293T cells expressing shRNA targeting TAg or an shRNA control (293T shControl) 48 h after co-transfection with the indicated pcDNA-based expression plasmids, assessed using a dual-luciferase reporter assay. Data are presented relative to cells transfected with an equivalent amount of pcDNA (±SD, n = 4). (D) Mean *Renilla* luciferase activity (mean relative light units ± SD, n = 4) from (C) measured as a control. (E) Culture supernatants from unmodified HEK293T cells or HEK293T cells expressing shTAg or shControl that had been transfected to produce a LV encoding GFP were harvested at 48 h and mean viral titers ± SD of biological replicates (n = 2) were determined in duplicate in HEK293T and THP-1 cells by enumerating GFP-positive cells. TU, transducing units.

We first depleted TAg expression in HEK293T by transduction with LV expressing TAg short hairpin RNA (shRNA) and selection in puromycin for 7 days. TAg mRNA levels were reduced by around 90% (Figure 4A) and TAg protein was reduced to levels that were not detected by immunoblot (Figure 4B). Rather than being more sensitive to activation, TAg-depleted cells had a slightly decreased response to cGAS/STING expression when compared to unmodified parental HEK293T or HEK293T expressing non-targeting shRNA (shControl cells) (Figure 4C). This was likely due to reduced expression of cGAS/STING because we also detected lower amounts of baseline *Renilla* luciferase expression from the control plasmid, which bears the SV40 early enhancer/promoter and therefore depends on TAg for amplification^42^ (Figure 4D). Thus luciferase and cGAS/STING expression is likely reduced due to reduced transfection efficiency or promoter activity. However, activation of the transfected promoter by co-transfected cGAS/STING was not greatly affected by reduction of TAg expression and loss of TAg certainly did not enhance innate activation by cGAS/STING in these experiments. Surprisingly, depletion of TAg had no significant effect on vector production yields when titer was measured either on HEK293T or THP-1 cells (Figure 4E).

**Figure 5:**
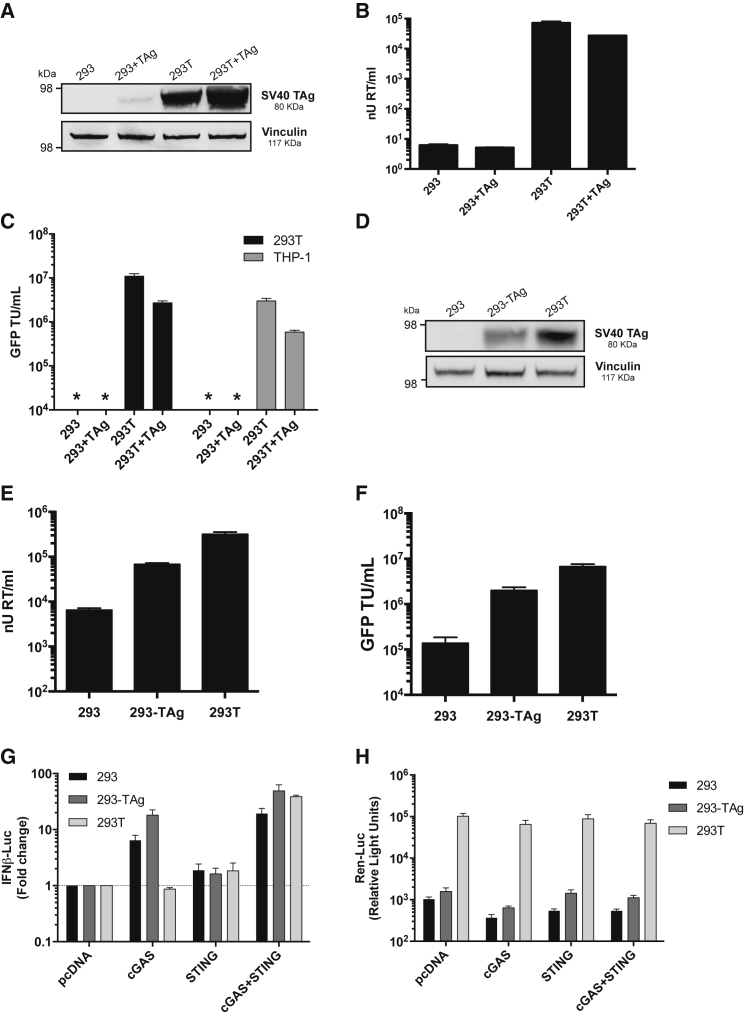
Large TAg Expression Enhances LV Production but Does Not Impact cGAS/STING-Driven Activity of the IFN-β Luciferase Reporter (A) TAg protein expression levels measured by immunoblot in unmodified HEK293 cells or in HEK293 or HEK293T after co-transfection of TAg encoding pcDNA. Molecular mass markers are indicated. Vinculin was detected as a loading control. (B) Measurement of LV encoding GFP (SG-PERT RT assay) produced in HEK293 or HEK293T cells co-transfected with empty vector or TAg encoding pcDNA (mean ± SD of biological replicates, n = 2). (C) Infectious titers of LV from (B) were evaluated in HEK293T or THP-1 cells by flow cytometry detecting GFP expression. Data are presented as mean transducing units (TU)/mL ± SD of biological replicates (n = 2) performed in duplicate. (D) Immunoblot detecting TAg protein levels in unmodified HEK293 or HEK293T cells, or HEK293 stably expressing TAg (293-TAg) from gammaretroviral vector pBABE-puro. Molecular mass markers are indicated, and vinculin was detected as a loading control. (E and F) Measurement of LV encoding GFP by (E) SG-PERT or (F) infection measuring GFP by flow cytometry, produced from unmodified HEK293 or 293T or 293 stably expressing TAg (293-TAg) (mean ± SD of biological replicates, n = 2). (G) Activation of IFN-β luciferase reporter in HEK293 (293), HEK293 cells stably expressing TAg-coding vector (293-TAg), or HEK293T cells (293T) after co-transfection with indicated pcDNA-based expression plasmids. Mean fold change in IFN-β activity (±SD, n = 4) was assessed 48 h after transfection and presented relative to cells transfected with an equivalent amount of empty pcDNA. (H) Mean *Renilla* luciferase activity (mean ± SD, n = 4) from (G) measured as a control.

In a second approach, we cloned the SV40 TAg cDNA into the expression vector pcDNA3 and co-transfected it with LV components in HEK293 or HEK293T (Figures 5A–5C). TAg is expected to amplify plasmids bearing an SV40 origin, including the HIV-1 packaging plasmid, leading to higher expression levels.^23^^,^^43^ TAg expressed from the pcDNA3 expression vector was detectable by western blot in HEK293 cells and at enhanced levels in HEK293T cells (Figure 5A). However, co-expression of TAg with LV components did not enhance LV production either in HEK293 or THP-1 cells (Figure 5B). LV production was measured by quantification of RT activity in cell supernatants by SYBR green PCR-enhanced reverse transcriptase assays (SG-PERT)^44^ (Figure 5B) or by infection of either HEK293T or THP-1 cells (Figure 5C). In fact titer from HEK293T cells transiently overexpressing TAg was slightly reduced (Figures 5B and 5C). We hypothesize that transient TAg expression in HEK293T does not improve LV production because HEK293T make sufficient TAg for maximum effect (Figure 5B). Detection of LV by SG-PERT assay but not by infection assay reflects the enhanced sensitivity of the PCR-based SG-PERT measurement.

Failure of co-transfected TAg to improve transfection efficiency could be due to the fact that TAg expression is required in cells at the time of transfection to drive the amplification of plasmids that contain the SV40 origin of replication,^42^ such as the HIV-1 packaging constructs and LV genome used in these experiments. We therefore stably expressed TAg using the gammaretroviral pBABE-puro SV40 TAg vector (Figures 5D–5H) followed by puromycin selection for 5 days. TAg expression levels in these cells was higher than the transiently expressing HEK293 (compare immunoblot in Figure 5A, transient expression, with that in Figure 5D, stable expression), but not as high as in the unmodified HEK293T cells (Figure 5D). We then transfected the modified cells (HEK293+TAg), and unmodified cells (HEK293 and 293T) with LV components to make LV-encoding GFP. LV production from unmodified HEK293 cells was low as measured by SG-PERT assay (Figure 5E) and infection (Figure 5F) but this was improved by TAg expression (HEK293+TAg), although not to the level of virus produced from unmodified HEK293T cells (Figure 5F).

The impact of stable TAg expression on IFN-β reporter activation by cGAS/STING expression was also minimal. Transfection of unmodified HEK293 or HEK293 stably expressing TAg with cGAS-encoding plasmid resulted in the activation of DNA sensing and a ∼5- to 10-fold induction of IFN-β reporter activity (Figure 5G), consistent with previous reports that HEK293 express functional STING.^45^ However, IFN-β reporter activity was slightly increased by TAg expression (Figure 5G), suggesting that TAg does not suppress inflammatory signaling downstream of DNA sensing. Rather we expect that TAg expression somewhat improves expression of cGAS/STING consistent with its enhancement of production of LV (Figures 5E and 5F). Consistent with this model, *Renilla* luciferase reporter activity in HEK293 cells stably expressing TAg were also slightly higher than in the unmodified HEK293 cells, although again, not as high as the unmodified HEK293T (Figure 5H).

Taken together, these data show that stable TAg expression does improve LV production when expressed in HEK293 that do not already express it, but that this is unlikely to be due to suppression of DNA sensing signals. Rather, TAg expression typically enhances expression from plasmids bearing an SV40 origin, as reported.^23^ It is not clear why TAg expression in cells that already make it (HEK293T, Figures 5B and 5C) reduces vector production, but it is possible that high levels of TAg expression are toxic.

## Discussion

In summary, we present data relevant to the production of therapeutic LV in HEK293T cells. We demonstrate that the HEK293T used to make LVs do not activate an IFN-β reporter after transfection of DNA likely in part because they do not express sufficient STING and cGAS (Figure 1D). However, the pathways downstream of STING activation are intact in HEK293T and cells transiently overexpressing exogenous STING and cGAS can activate ISG induction, although this does not particularly reduce LV production or the infectivity of produced LV. Furthermore, inflammatory cytokines type 1 IFN and CXCL10 are not found in measurable amounts in 48 h supernatants from activated HEK293T cells. Certain transgenes, exemplified here by CAR and TRIM5-CypA, can activate inflammatory transcriptional responses, particularly NF-κB, but these are only associated with minor, if any, reduction in LV production. Importantly we show that, should type 1 IFN be produced during LV production, this does not impact infectivity of VSV-G-pseudotyped LVs on peripheral blood mononuclear cells (PBMCs), a key primary target of LV therapy.

The protocols we have used herein to make LVs differ somewhat to those used most recently to produce clinical grade LVs at large scale. For example, clinical LV production typically uses calcium-phosphate-based transfection protocols followed by purification of the LVs by column chromatography with a view to remove HEK293T products including inflammatory cytokines. Experiments presented here suggest that purification from inflammatory cytokines may not be necessary. Furthermore, we have used a 2^nd^ generation packaging plasmid (pCMV-dR8.74) encoding Gag-Pol, Tat, and Rev, whereas more recent gene therapy protocols use a 3^rd^ generation system, which has eliminated Tat and split packaging functions further. 3^rd^ generation packaging systems express Rev on a separate plasmid and use a Tat-independent expression cassette for Gag-Pol. This is expected to be less likely to recombine to make infectious virus, although infectious recombinant virus has never been described from a 2^nd^ generation system, likely due to the lack of an *Env* gene. One reason to test a 2^nd^ generation system is the higher expression levels of viral proteins achieved due to increased transfection efficiency of fewer plasmids, maximizing opportunity for innate immune activation. Importantly, the LV components expressed are the same in both systems with the exception of Tat, which is not included in 3^rd^ generation systems. We propose that 3^rd^ generation systems are even less likely to induce a response due to viral proteins. However, our data show that induction of innate sensing by vector components is small, and innate immune related transgenes can activate innate sensors more effectively; for example, TRIM5α. Overall, our data suggest that HEK293T transfection does not induce effective antiviral responses that limit LV production and any responses activated have minimal impact on target cell transduction, as shown.

Finally, we made the surprising observation that depleting TAg antigen expression from HEK293T did not particularly reduce LV production. However, stable expression of TAg in HEK293 cells significantly improved their ability to produce LV by transient transfection. We hypothesize that TAg expression contributes directly to the production of LV from plasmids encoding a TAg origin as reported and that TAg expression over time may have led to further adaptation of HEK293T cells to become particularly efficient for LV production. We also found that TAg did not have any particular inhibitory activity against innate immune activation downstream of DNA sensing driven by cGAS and STING expression in HEK293T cells. Together these observations are consistent with the empirical establishment of HEK293T cells as effective producers of therapeutic LVs and provide understanding of mechanisms underlying this process.

## Materials and Methods

### Cell Culture

HEK293T cells used in this study were previously generated by the Rayne Cell Therapy Suite (King’s College London) for clinical LV production^46^ and were kindly provided by Farzin Farzaneh. They are derived from the 293T/17 cell line obtained from ATCC (CRL-11268). HEK293FT cells were obtained from Thermo Fisher Scientific and HEK293T JL cells were kindly provided by the laboratory of Jeremy Luban (University of Massachusetts Medical School, Worcester, MA, USA). All HEK293T cells were cultured in Dulbecco’s modified Eagle’s medium (DMEM; Thermo Fisher Scientific, Waltham, MA, USA). Human THP-1 cells were grown in RPMI 1640 medium (Thermo Fisher Scientific). All media were supplemented with 10% heat inactivated fetal calf serum (FCS) and penicillin-streptomycin (50 μg/mL; Thermo Fisher Scientific) and cell cultures were maintained at 37°C in a 5% CO_2_ incubator. When indicated, IFN-β (PeproTech, Rocky Hill, NJ, USA) was added to cells at 0.1–1,000 ng/mL (28 − 2.8 × 10^5^ U/mL) 2 h before transfection or transduction. IFN-β was always supplemented at the time of medium replacement.

### Reporter Gene Assays

For reporter gene assays, cells were seeded 24 h prior to transfection at the appropriate density in 6-well plates when followed by LV titration or in 48-well plates otherwise. Cells were transfected using Fugene HD (Promega), according to the manufacturer’s protocol with reporter plasmids encoding promoters based on various genes or synthetic constructs driving expression of luciferase (luc). These included the IFN-β promoter (Promega), a synthetic promoter bearing 5 NF-κB p50/p65 binding sites (Promega), the IgK3conAluc plasmid (containing three copies of the Igκ chain enhancer NF-κB binding site upstream of the conalbumin promoter^47^), the IFIT1 promoter (kindly provided by Geoffrey Smith, University of Cambridge, UK) and pRL-TK *Renilla* luciferase plasmid (Promega). Empty pcDNA3.1 plasmid was used to equalize DNA amounts between wells. Cells were lysed in passive lysis buffer (Promega) 48 h post transfection and Firefly and *Renilla* luciferase activities were measured using a Dual-Luciferase Assay (Promega) and a FLUOstar OPTIMA luminometer (BMG Labtech, Ortenberg, Germany), following manufacturer’s instructions. Firefly luciferase activity was normalized to the *Renilla* luciferase activity and the fold induction of each reporter activity was calculated by normalizing each result to that of the control cells transfected with empty vector. Where indicated, JAK inhibitor ruxolitinib (2 μM, Cell Guidance Systems) was added 24 h post transfection. Gaussia luciferase activity was measured using coelenterazine substrate (Sigma-Aldrich, St. Louis, MO, USA) and a FLUOstar OPTIMA luminometer (BMG Labtech).

### LV Production

HEK293T were co-transfected with transfer vector construct (SFFV-eGFP, SFFV-TRIMCyp, or PGK-CAR),^36^ pCMV-dR8.74 or pCMV-dR8.91 (packaging plasmids encoding Gag-Pol, Tat, and Rev, Addgene #22036) and pMD2.G (VSV-G envelope expression plasmid,^48^ Addgene #12259) as described previously.^10^ Briefly, cells were seeded 24 h before transfection using Fugene HD (Promega) according to the manufacturer’s protocol. Media were changed 24 h later and LV-containing media were collected 48 h post transfection, passed through a 0.45 μm filter, and stored at −80°C.^49^ In experiments investigating the role of TAg, we co-transfected 500 ng of pcDNA3 encoding TAg (or empty pcDNA3) with 333 ng HIV-1 packaging plasmid, 333 ng VSV-G encoding plasmid, and 500 ng GFP bearing HIV-1 genome plasmid into a well of a 6-well plate of HEK293 cells and harvested supernatants at 48 h post transfection. To stably express TAg, we transduced HEK293 cells on a 10 cm dish with murine leukemia virus (MLV) particles packaging pBABE puro encoding TAg. 48 h after transfection, we added 1 μg/mL of puromycin. Cells were selected for 5 days and then used to make LVs as described above.

### LV Titer Measurements

Vector titers were determined as described previously^50^ by adding serial dilutions of LV to 6 × 10^5^ HEK293T or THP-1 cells seeded per well in a 12-well plate 24 h before transduction. Polybrene (Sigma-Aldrich) was added to the medium at a final concentration of 8 μg/mL. For GFP-encoding vectors, cells were analyzed by flow cytometry 3 days post-transduction and the percentage of GFP-expressing cells used to calculate the number of transducing units (TU) per mL of vector. For LV-CAR, titer was determined using a TaqMan assay. Briefly, HEK293T cells were transduced as described and passaged every 3–4 days for a total of 10 days. Genomic DNA extraction was performed using the QIAamp DNA Blood Mini Kit (QIAGEN). LV DNA was measured using specific probes for the vector packaging sequence (ψ) and for the cellular albumin gene (Table S1),^51^ and values were compared to a standard curve to determine the number of transducing units per mL of vector.

### Primary Cell Titer Measurements

PBMCs were isolated by Ficoll (GE Healthcare) gradient, resuspended in X-VIVO 15 supplemented with 5% human AB sera (Lonza) and 100 international units of interleukin-2 (IL-2), and activated at a 1:1 ratio with human T-Activator CD3/CD28 Dynabeads (Thermo Fisher Scientific). LV-CAR transduction efficiency was assessed by flow cytometry staining cells with biotin AffiniPure F(ab’) fragment goat anti-mouse immunoglobulin IgG (Jackson Immunoresearch, West Grove, PA, USA) followed by streptavidin-APC (Biolegend, San Diego, CA).

### CXCL10 ELISA

Cell culture supernatants from HEK293T cells grown in 6-well plates were assayed at 48 h post transfection for CXCL10 protein using Duoset enzyme-linked immunosorbent assay (ELISA) reagents (R&D Systems) according to the manufacturer’s instructions. THP-1 cells were stimulated with cGAMP for 24 h as a positive control (1 μg/mL, Invivogen).

### Quantitative Reverse Transcriptase PCR (qRT-PCR)

Total RNA was extracted from cells using RNeasy RNA extraction kit (QIAGEN N.V., Hilden, Germany) according to the manufacturer’s protocol. Complementary DNA was synthesized using SuperScript III reverse transcriptase (Invitrogen, Carlsbad, CA, USA) with 500 ng of RNA and following the manufacturer’s instructions. cDNA was diluted 5-fold in water, and 2 μL were used as the template for real-time PCR using Fast SYBR green PCR master mix (Applied Biosystems, Foster City, CA, USA). Expression of each gene was normalized to that of an internal control (GAPDH), and these values were then normalized to the value of control cells to yield the fold change. All qPCR reactions were performed in technical and biological duplicates. Primer sequences can be found in Table S2.

### Immunoblots

Cell extracts were prepared by washing 5 × 10^6^ cells with cold PBS and resuspending cell pellets in radioimmunoprecipitation assay (RIPA) buffer supplemented with Halt protease inhibitor cocktail (Thermo Fisher Scientific). Whole cell lysis was carried out on ice for 15 min and lysates cleared by centrifugation in a tabletop centrifuge at full speed for 5 min. Total protein was quantified using BCA Protein Assay Reagents (Pierce Thermo Fisher Scientific) and 25 μg total protein extract was heated at 95°C for 5 min with NuPAGE LDS Sample Buffer (Thermo Fisher Scientific) and then loaded on a Novex NuPAGE 4%–12% Bis-Tris gel (Thermo Fisher Scientific). Wet transfer was performed using the X-Cell SureLock Blot module (Thermo Fisher Scientific) onto Immun-blot PVDF Membrane (BioRad, Hercules, CA, USA). Membranes were blocked with 5% skimmed milk in PBS-Tween for 1 h at room temperature and incubated overnight at 4°C with the primary antibody. Membranes were washed the following day 3 times for 10 min and incubated with HRP-conjugated secondary antibody for 1 h. After washing, membranes were incubated with Super Signal West Pico Chemiluminescent Substrate (Thermo Fisher Scientific) and visualized using UviChemi Chemiluminescence Documentation System. Primary antibodies used were rabbit anti-cGAS mAb (Cell Signaling Technology #15102, Danvers, MA, USA), mouse anti-STING (Novus Biologicals MAB7169, Littleton, CO, USA), mouse anti-SV40 TAg antibody (ab16879, Abcam, Cambridge, UK), and mouse anti-vinculin (Sigma-Aldrich SAB4200080). The secondary antibodies used were donkey anti-rabbit IgG-HRP (GE Healthcare NA934V, Chicago, IL, USA) and sheep anti-mouse IgG-HRP (GE Healthcare NXA931).

### shRNA-Mediated Depletions

For lentiviral expression, HEK293T were transfected as described^49^ with pHR-SIREN constructs expressing shRNA-encoding oligonucleotides and the packaging plasmids pCMV-dR8.74 and pMD2.G. Target cells were transduced at MOI of 2 and selected in 2.0 μg/mL puromycin 72 h later for 5–7 days. The 19-mer target sequences are listed in Table S3.

### Plasmid Construction

To clone SV40 TAg cDNA, we extracted total RNA from HEK293T cells and synthesized cDNA as described above. SV40 TAg coding DNA sequence (GenBank: J02400.1) was amplified by PCR using Q5 High Fidelity Polymerase (New England Biolabs, Ipswich, MA, USA) and primers are presented in Table S4. The PCR fragment was cloned into EcoRI-NotI linearized pcDNA3.1 (Thermo Fisher Scientific) using T4 DNA ligase (New England Biolabs) following manufacturer’s instructions.

### SG-PERT

Reverse transcriptase activity in diluted cell supernatants was quantified by qPCR using a SYBR green-based product-enhanced RT (SG-PERT) assay as described.^52^

## Author Contributions

Conceptualization, C.B.F., R.P.S., W.Q., and G.J.T.; Methodology, C.B.F. and R.P.S.; Investigation, C.B.F., R.P.S., and M.T.R.-P.; Validation, R.P.S.; Formal Analysis, C.B.F.; Visualization and Writing – Original Draft Preparation, C.B.F., R.P.S., and M.T.R.-P.; Writing – Review & Editing, C.B.F., R.P.S., J.R., R.S.M., W.Q., and G.J.T.; Project Administration, C.B.F.; Supervision, G.J.T. and W.Q.; Funding Acquisition, C.B.F., A.J.T., W.Q., and G.J.T.

## Conflicts of Interest

Unrelated to this study, W.Q. holds equity in Autolus Ltd and Orchard Therapeutics.

## Supplemental Information

Document S1. Figures S1 and S2 and Tables S1–S4

Document S2. Article plus Supplemental Information
