# Supplementary material for: Lentiviral Vector Production Titer Is Not Limited in HEK293T by Induced Intracellular Innate Immunity
Source: Mol Ther Methods Clin Dev. 2019 Dec 24;17:209–19. doi: 10.1016/j.omtm.2019.11.021 (PMC6965512; doi:10.1016/j.omtm.2019.11.021)
Supplement: Document S1. Figures S1 and S2 and Tables S1–S4 [file mmc1.pdf]

OMTM, Volume *17*

Supplemental Information

Lentiviral Vector Production Titer Is Not Limited in HEK293T by Induced Intracellular Innate Immunity

Carolina B. Ferreira, Rebecca P. Sumner, Maria T. Rodriguez-Plata, Jane Rasaiyaah, Richard S. Milne, Adrian J. Thrasher, Waseem Qasim, and Greg J. Towers

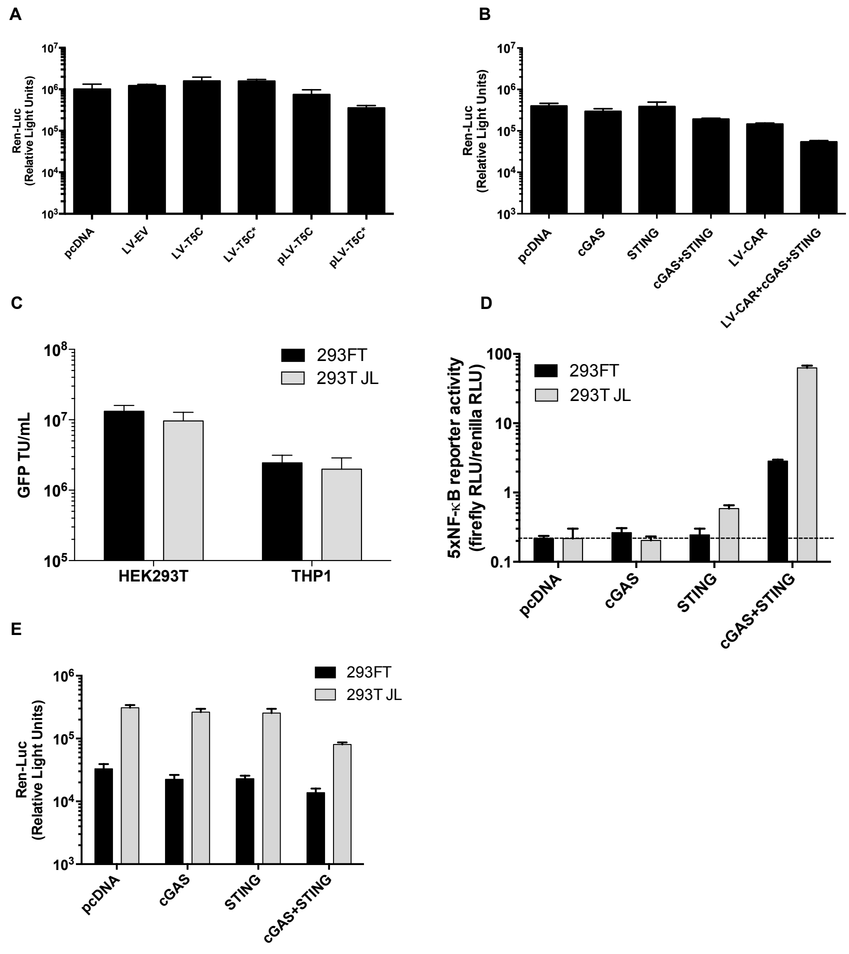

Figure S1. Production of gene therapy LV in HEK 293T triggers NF-κB activation but does not impact LV yield.

(A) *Renilla* luciferase readings for reporter gene assay data presented in Figure 1A (IgK reporter assay). HEK 293T were transfected with firefly luciferase reporter constructs, pRL-TK *Renilla* luciferase and empty pcDNA3 as a control or lentiviral vector constructs as described in Figure 1A. Luciferase activity was measured at 48 h post transfection. Data are presented as mean luminometry readings (relative lights units) ± s.d. **(B)** *Renilla* luciferase readings for reporter gene assay data presented in Figure 1C (IgK reporter assay). HEK 293T were transfected with firefly luciferase reporter constructs, pRL-TK *Renilla* luciferase and empty pcDNA3 as a control or lentiviral vector constructs as described in Figure 1C. Luciferase activity was measured at 48 h post transfection. Data are presented as mean luminometry readings (relative lights units) ± s.d. **(C)** Culture supernatants from HEK 293FT or HEK 293T JL cells that had been transfected to produce a GFP-encoding LV were harvested at 48 h and mean viral titres ± s.d of biological replicates (n = 2) were determined in quadruplicate in HEK 293T and THP-1 cells by enumerating GFP-positive cells. **TU** transducing units. **(D)** HEK 293FT or HEK 293T JL cells were transfected with 5xNF-κB firefly luciferase reporter construct, pRL-TK *Renilla* luciferase and pcDNA-based expression plasmids encoding cGAS and/or STING as shown. Mean reporter activity ± s.d (n = 2) was assessed 48 h later using a dual-luciferase reporter assay and is presented as firefly luciferase relative light units (RLU) divided by *Renilla* luciferase RLU. **(E)** *Renilla* luciferase readings for reporter gene assay data presented in Figure S1D.

A

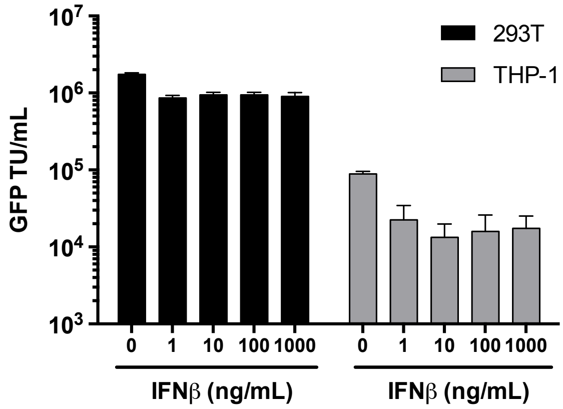

Figure S2. Exogenous IFNβ reduces LV transduction efficiency on monocytic cells but not on HEK 293T or primary T cells.

HEK 293T cells were used to produce GFP-encoding lentiviral vector in the absence or presence of increasing amounts of IFNβ. This experiment simulates the situation in which HEK 293T cells make IFN as a by-product of lentiviral vector production and secrete it into the LV prep. Culture supernatants were harvested at 48 h post transfection and viral titres ± s.d. of biological replicates (n = 2) were determined in duplicate by FACS of GFP-positive HEK 293T or THP-1 cells.

Supplementary Table 1. Taqman primers and probes

Primer/Probe	Sequence (5' – 3')	Reference
Psi Fwd	CAGGACTCGGCTTGCTGAAG	Schott et al, 2019[1]
Psi Rev	TCCCCCGCTTAATACTGACG	Schott et al, 2019[1]
Psi Probe	FAM-CGCACGGCAAGAGGCGAGG-TAMRA	Schott et al, 2019[1]
Albumin Fwd	GCTGCTATCTCTTGTGGGCTGT	Schott et al, 2019[1]
Albumin Rev	ACTCATGGGAGCTGCTGGTTC	Schott et al, 2019[1]
Albumin Probe	VIC-CCTGTCATGCCCACACAAATCTCTCC-TAMRA	Schott et al, 2019[1]

Supplementary Table 2. Real time PCR primers

Primer	Sequence (5' – 3')	Reference
hGAPDH Fwd	ACCCAGAAGACTGTGGATGG	Stuart et al, 2016[2]
hGAPDH Rev	TTCTAGACGGCAGGTCAGGT	Stuart et al, 2016[2]
IFNβ Fwd	AGGACAGGATGAACTTTGAC	Gao et al, 2013[3]
IFNβ Rev	TGATAGACATTA GCC AGGAG	Gao et al, 2013[3]
CXCL10 Fwd	TGGCATTCAAGGAGTACCTC	Gao et al, 2013[3]
CXCL10 Rev	TTGTAGCAATGATCTCAACACG	Gao et al, 2013[3]
ISG56 Fwd	CCTCCTTGGGTTCGTCTACA	Jakobsen et al, 2013[4]
ISG56 Rev	GGCTGATATCTGGGTGCCTA	Jakobsen et al, 2013[4]
ISG54 Fwd	CAGCTGAGAATTGCACTGCAA	Jiang et al, 2010[5]
ISG54 Rev	CGTAGGCTGCTCTCCAAGGA	Jiang et al, 2010[5]
TAg Fwd	TGAGGCTACTGCTGACTCTCAACA	Bergsagel et al, 1992[6]
TAg Rev	GCATGACTCAAAAAACTTAGCAATTCTG	Bergsagel et al, 1992[6]

Supplementary Table 3. SV40 Large T antigen cloning primers

Primer	Sequence (5' – 3')	Reference
EcoRI-TAg Fwd	GCATGAATTCATGGATAAAGTTTTAAACAGAGAGG	This study
TAg-NotI Rev	ATGCGGCCGCTTATGTTTCAGGTTCAGGGGG	This study

Supplementary Table 4. shRNA target sequences

shRNA	shRNA target sequence (5' – 3')	Reference
shTAg	TGGGCAACAAACAGTGTAG	This study
shControl	ATGTCTCTGGAAAAGATGT	This study

SUPPLEMENTAL REFERENCES

1. Schott, J. W. *et al.* Enhancing Lentiviral and Alpharetroviral Transduction of Human Hematopoietic Stem Cells for Clinical Application. *Molecular therapy. Methods & clinical development* **14**, 134-147, doi:10.1016/j.omtm.2019.05.015 (2019).
2. Stuart, J. H., Sumner, R. P., Lu, Y., Snowden, J. S. & Smith, G. L. Vaccinia Virus Protein C6 Inhibits Type I IFN Signalling in the Nucleus and Binds to the Transactivation Domain of STAT2. *PLoS Pathog* **12**, e1005955, doi:10.1371/journal.ppat.1005955 (2016).
3. Gao, D. X. *et al.* Cyclic GMP-AMP Synthase Is an Innate Immune Sensor of HIV and Other Retroviruses. *Science* **341**, 903-906, doi:10.1126/science.1240933 (2013).
4. Jakobsen, M. R. *et al.* IFI16 senses DNA forms of the lentiviral replication cycle and controls HIV-1 replication (vol 110, pg E4571, 2013). *Proceedings of the National Academy of Sciences of the United States of America* **110**, 19651-19651, doi:10.1073/pnas.1320190110 (2013).
5. Jiang, L., Saetre, P., Radomska, K. J., Jazin, E. & Lindholm Carlstrom, E. QKI-7 regulates expression of interferon-related genes in human astrocyte glioma cells. *PloS one* **5**, doi:10.1371/journal.pone.0013079 (2010).
6. Bergsagel, D. J., Finegold, M. J., Butel, J. S., Kupsky, W. J. & Garcea, R. L. DNA sequences similar to those of simian virus 40 in ependymomas and choroid plexus tumors of childhood. *N Engl J Med* **326**, 988-993, doi:10.1056/NEJM199204093261504 (1992).
